# Parental Somatic Mosaicism Detected During Prenatal Diagnosis

**DOI:** 10.1002/pd.6712

**Published:** 2024-11-25

**Authors:** Natalie J. Chandler, Elizabeth Scotchman, Fiona McKay, Vijaya Ramachandran, Lyn S. Chitty

**Affiliations:** ^1^ NHS North Thames Genomic Laboratory Hub Great Ormond Street Hospital for Children NHS Foundation Trust London UK; ^2^ Genetics and Genomic Medicine UCL Great Ormond Street Institute of Child Health London UK

## Abstract

**Objective:**

Accurate recurrence risks are essential for genomic counselling and parental reproductive choices. Historically, Sanger sequencing was used to test parental samples, which has a limited sensitivity of ∼ 10% for detecting somatic mosaicism. Next generation sequencing (NGS) methods, utilised for non‐invasive prenatal diagnosis (NIPD) and trio prenatal exome sequencing in our laboratory, have greater sensitivity. Here we review the cases of parental somatic mosaicism we have detected and discuss its impact on management.

**Method:**

Laboratory databases from 1 January 2015 to 30 September 2022 were reviewed to identify all cases where parental somatic mosaicism was detected during NIPD and prenatal exome testing.

**Results:**

During the development of NIPD testing, we identified 10/131 (7.6%) families with parental somatic mosaicism. In six cases where NGS detected levels between 0.37% and 8.82%, prior testing with Sanger sequencing had not detected mosaicism. In our exome sequencing cohort, we detected parental mosaicism in 4/101 (3.96%) cases. Clinical features of the condition were identified in 2/14 parents.

**Conclusion:**

The sensitivity of the testing technique needs to be considered when counselling parents on recurrence risk. Parents need to be aware that modern approaches to prenatal diagnosis may allow identification of mosaicism, which may have implications for their own health and change recurrence risks for future pregnancies.

**Summary**

- What's already known about this topic?
  ◦ Multiple sequencing technologies are utilised for prenatal diagnosis of single gene disorders and the sensitivity of each method to detect parental somatic mosaicism is variable.
  ◦ Identification of parental somatic mosaicism impacts recurrence risk of future pregnancies.
- What does this study add?
  ◦ This study highlights that the sensitivity of the testing method needs to be considered when counselling parents on recurrence risk and that laboratories need to provide information regarding this in reports.
  ◦ It demonstrates the need to test the maternal genomic DNA sample alongside the cell‐free DNA for all NIPD/S assays to avoid the possibility of maternal mosaicism leading to false positive results.

## 1 Introduction

Accurate recurrence risks are essential for genomic counselling and parental reproductive choices. Prior to the introduction of next generation sequencing (NGS) technologies, parents of a child with a rare de novo dominant condition were conventionally tested for the pathogenic variant using Sanger sequencing. If the variant is not detected in the parental DNA tested then parents are counselled on the basis of a germline mosaicism risk which is usually very low [1]. In cases where somatic mosaicism is found, the recurrence risk can approach 50% depending upon the level of parental mosaicism [2, 3]. Accurate recurrence risk also has implications for testing options for future pregnancies; for example the NHS in England will only fund PGT‐M for recurrence risks above 10% [4] and non‐invasive prenatal diagnosis is only funded where the father is known to carry the variant (including when he is a somatic mosaic). Sanger sequencing has limited sensitivity and only detects somatic mosaicism down to ∼ 10% (ref. [5] and unpublished data from our laboratory). With advances in technologies following the introduction of next generation sequencing, numerous studies have reported parental somatic mosaicism that has been missed by Sanger sequencing [4, 5, 6, 7, 8]. Despite these studies, Sanger sequencing has continued to be used for testing parental samples when the diagnosis has been established using singleton NGS analysis.

The adoption of NGS technologies has revolutionised prenatal genetic testing services [9]. In 2012, our laboratory began offering an accredited non‐invasive prenatal diagnosis (NIPD) service for families at known increased risk of monogenic disorders, either because of a family history or sonographic findings. Initially this was confined to *FGFR2* and *FGFR3*‐related disorders [10, 11] for which we have tested over 900 pregnancies [12]. In 2014, the service was expanded to offer paternal exclusion in pregnancies at risk of recessive disorders where parents carried different pathogenic variants [13], and from 2016 we began offering definitive NIPD for recessive disorders taking account of the maternal pathogenic variant and where parents carried the same pathogenic variant [14]. In 2015, the range of NIPD tests offered was broadened to include a bespoke NIPD service to test for family‐specific paternally inherited pathogenic variants or apparently de novo pathogenic variants in a previous child. Our method involves PCR amplification of the variant of interest followed by next generation sequencing. In‐house studies have shown this method to have a sensitivity of ∼ 1% [15], making it suitable for detecting foetal variants against the maternal background in cell free DNA. As part of the development of bespoke NIPD for individual families, each assay is validated using genomic DNA samples from both parents and probands. During this process, we identified some cases of unexpected parental somatic mosaicism.

In addition to our NIPD services, we have expanded our prenatal testing to offer rapid trio exome sequencing for foetuses with anomalies detected by imaging [16]. Our laboratory is one of the two centres offering this publicly funded service in England. The sensitivity of exome sequencing for detecting parental somatic mosaicism is variable as it is dependent on both the depth of sequencing reads at the position of the variant and the filtering of the bioinformatics pipeline [17]. However, numerous studies have reported the detection of parental somatic mosaicism when trio exome testing is performed [3, 7, 16, 17, 18, 19, 20].

The objective of this study was to review laboratory databases for our NIPD and prenatal trio exome sequencing services to identify cases with parental somatic mosaicism. We investigated the extent of parental mosaicism and its implications for families and service delivery.

## 2 Methods

### 2.1 Audit of Service

Laboratory databases were reviewed (prior to September 2022) to identify all cases referred for bespoke NIPD assay development where the father was not a heterozygous carrier of the variant, and for prenatal trio exome sequencing to ascertain those where parental somatic mosaicism was detected during testing. Where detected, we performed Sanger sequencing to determine if this was able to detect the mosaicism. We also contacted the referring clinicians to ask if the parent with mosaicism had any phenotypic signs of the condition after clinical review.

### 2.2 NIPD Testing and Calling of Mosaicism

Details of the NIPD methodology have been previously described [10]. Briefly, for family specific (bespoke) NIPD a single amplicon targeting the variant of interest is amplified. DNA extracted from blood samples from both parents and affected probands were tested in parallel and sequenced using an Illumina MiSeq. After sequencing, the number of reads containing a sequence specific for either the wild‐type or variant allele and 10 base pairs on either side were counted for each target site using an in‐house bioinformatics pipeline. The percentage variant allele frequency (VAF) was calculated for each of the parental gDNAs. Normal controls were also run to assess the background variant level, which must be under 0.12% (see supplementary data). Any variant value over 1% was automatically classed as a somatic mosaic. For the case under 1% (0.35%), the testing was performed 3 times to ensure the result was reproducible, which allowed us to be confident in calling the sample as somatic mosaic. See Supporting Information S1 (Assessing background levels and lower limit of detection section) for how these thresholds were determined.

### 2.3 Prenatal Exome Testing and Calling of Mosaicism

Details of the prenatal exome sequencing methodology have been previously described [16]. Briefly, exome capture was performed on DNA samples extracted from the proband and both parents (blood samples) and sequenced using an Illumina Nextseq. The foetal anomalies panel [21] was applied bioinformatically and variant filtering and prioritisation was performed prior to variant classification. The bioinformatics pipeline only calls variants at a VAF over 10% and with a minimum of five alternate reads. Any variants that were heterozygous in the foetus and under 35% VAF in one of the parents were deemed to be mosaic. Parental sequencing reads were inspected manually for all cases that had a de novo variant in the foetus and somatic mosaicism was called when present in two or more sequencing reads in one of the parents.

### 2.4 Sanger Sequencing

When somatic mosaicism was detected, Sanger sequencing using conventional methods was performed to determine if the variant could be detected. Briefly, the region containing the variant was amplified and then sequenced bidirectionally using an ABI 7300XL instrument. Sequencing traces were compared against the reference sequence for the relevant gene and a known normal control manually.

## 3 Results

**FIGURE 1:**
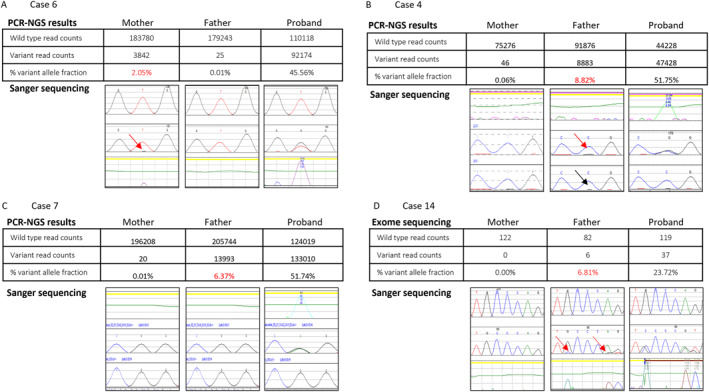
A comparison of Sanger sequencing and PCR capture next generation sequencing results of parental and affected proband genomic DNA samples for selected cases ((A)—Case 6; (B)—Case 4; (C)—Case 7; (D)—Case 14); see Supporting Information S1: Figures 1–3 for all cases. For the Sanger sequencing traces, all variants occur at the middle peak in each image. The middle panel in each figure shows traces from the sample tested, the other sequence trace is from a normal control sample (either top or bottom depending on if forward or reverse) and the other panel is the electropherogram traces comparing the peaks detected in the sample trace in comparison to the normal control. Red arrows indicate signs of mosaicism and black arrows indicate background noise causing mosaicism to be missed. PCR capture Next Generation Sequencing results are given as the number of reads for the wild type and variant sequences. The percentage VAF has also been calculated using the values in red indicating somatic mosaicism.

Prior to September 2022, a total of 131 families where the father was not a heterozygous carrier underwent successful family specific NIPD assay development. Parental somatic mosaicism was detected in 10 of these (7.6%) (Table 1), four maternal and six paternal. In cases 1–6, parental mosaicism was not known prior to the referral for NIPD testing (Table 1). The NIPD assay (PCR‐NGS) results and Sanger sequencing traces can be seen in Supporting Information S1: Figure 1A–F. The levels of somatic mosaicism detected ranged from 0.37% to 8.82%. For five of the cases, where the variant was heterozygous in the proband, the percentage VAF was close to the expected value of 50% (mean: 50.4, range: 45.56%–51.77%). For the hemizygous proband, the percentage VAF was again close to the expected value of 100% (99.8%). These results show that the assays detected the variant at the expected level. To assess the background level, normal controls were run with the highest background seen at 0.06%, further confirming the assays were of good quality and met our quality control criteria. For 5/6 of these cases, parental testing had been performed by Sanger sequencing prior to referral for NIPD on DNA extracted from blood. The method of testing was unknown for case 5. Sanger sequencing for validation of the parental somatic mosaicism showed no evidence of the variant in the parental samples for cases 1, 2, 3 and 5. For case 6 (Figure 1A), the variant is visible at low level and for case 4 (Figure 1B) the variant is present at low level but there is background noise in the normal control sample and therefore the analyst did not call the mosaicism.

**TABLE 1:** Summary of the cases where parental somatic mosaicism was detected during NIPD assay development. For cases 1–6, the parental mosaicism was not known prior to referral. For cases 7–10 the parental mosaicism was known prior to referral.

Case	Gene and variant	Condition	Technique used to test parental samples	Level of mosaicism (%)	Parent with mosaicism	NIPD tests	Clinical follow‐up of parent
1	*OTC* NM_000531.6 c.521C>T p.(Ala174Val)	Ornithine transcarbamylase deficiency	Sanger	2.71	Mother	n/a	No symptoms
2	*COL1A1* NM_000088.4 c.544‐1G>A p.?	Osteogenesis imperfecta	Sanger	3.88	Mother	n/a	No symptoms
3	*COL1A1* NM_000088.4 c.941G>A p.(Gly314Glu)	Osteogenesis imperfecta	Sanger	4.73	Mother	n/a	No symptoms
4	*FGFR3* NM_000142.5 c.779C>G p.(Pro260Arg)	Hypochondroplasia (rare variant)	Sanger—visible on sanger trace but sequencing not clean	8.82	Father	1x not affected	Normal stature, no features
5	*COL1A1* NM_000088.4 c.1597G>A p.(Gly533Ser)	Osteogenesis imperfecta	Unknown	0.37	Father	1x not affected	No symptoms
6	*TUBB4A* NM_006087.4 c.938T>G p.(Val313Gly)	Hypomyelinating leukodystrophy type 6	Sanger	2.05	Mother	n/a	No symptoms
7	*COL1A2* NM_000089.4 c.1801G>A p.(Gly601Ser)	Osteogenesis imperfecta	Pyrosequencing	6.37	Father	1x affected	No symptoms
						2x not affected	
8	*FBN1* NM_000138.5 c.8268G>A p.(Trp2756*)	Marfan syndrome	Sanger on genomic DNA in blood and buccal cells	12.04	Father	1x not affected	Normal phenotype, echocardiogram normal
9	*TSC2* NM_000548.5 c.2309_2313dup p.(Ala772*)	Tuberous sclerosis	Suspected by sanger sequencing on blood, confirmed by COLD PCR in brain tissue	7.95	Father	1x not affected	Father clinically affected
10	*NOTCH2* NM_024408.4 c.6909dup p.(Ile2304Hisfs*9)	Hajdu–Cheney syndrome	Trio exome sequencing	22.41	Father	Not had NIPD yet	No symptoms

Abbreviation: n/a, not applicable.

Following clinical review, none of the somatic mosaic parents identified by NIPD assay development had any clinical features suggestive of the condition in question. In the two cases of paternal mosaicism, NIPD was offered with both the foetuses found to be unaffected. We were unable to offer NIPD where maternal somatic mosaicism was detected in the mother as we are unable to distinguish between the maternal and foetal cell free DNA.

For cases 7–10, paternal somatic mosaicism was known prior to referral for NIPD development (Table 1). The NIPD assay (PCR‐NGS) results and Sanger sequencing traces can be seen in Supporting Information S1: Figure 2A–D. The levels of somatic mosaicism detected ranged from 6.37% to 22.41%. In case 7 (Figure 1C), paternal mosaicism was not identified using Sanger sequencing but was identified using pyrosequencing as parental mosaicism was suspected due to having two previously affected pregnancies. For cases 8 and 9, mosaicism was suspected from Sanger sequencing and confirmed by testing alternative tissue types. For case 10, where the mosaic level by PCR‐NGS was at the highest level (22.41%), mosaicism was detected by trio exome sequencing. Clinical features were only present in case 9 as the father was the proband in this case. NIPD tests have been performed for 3/5 of these pregnancies with one pregnancy found to be affected and four unaffected.

For the prenatal trio exome sequencing cohort prior to September 2022, autosomal dominant conditions were diagnosed in 101 foetuses. Of these 6 (5.9%) were inherited from a parent that was also heterozygous for the variant and in 4 (4.0%) cases one of the parents was unavailable for testing leaving 91 where both parental samples were available and neither parent was heterozygous for the variant. In four of these 91 cases (4.4%), parental somatic mosaicism was detected. The parental somatic mosaicism ranged from 6.77% to 26.09% (cases 11–14—Table 2). The exome sequencing results and Sanger sequencing traces are shown in Supporting Information S1: Figure 3A–D. As expected from the pipeline settings, the two cases with parental mosaicism over 10% were called by the pipeline as being present in the mosaic parent. The two cases below 10% were not called by the pipeline and the mosaicism was detected by manual inspection of the sequencing reads. The sequence read alignments are shown in Supporting Information S1: Figures 4–7. The pathogenic variant in case 14 was a 17bp deletion and has only been called in 23.72% of reads in the proband, below the expected 50%. Due to the challenges in calling small insertions and deletions (indels) of this size from short read sequencing, it is likely that this variant is truly heterozygous and therefore the percentage VAF in the father is also likely to be underestimated. Sanger sequencing results for this case are in support of the proband being heterozygous (Figure 1D). Clinical features were only present in the somatic mosaic parent in case 12, where the mother presented with milder features of a *COL2A1*‐related disorder whereby the foetus presented with a lethal phenotype.

**TABLE 2:** Summary of the four cases where parental somatic mosaicism was detected during prenatal trio exome sequencing.

Case	Gene and variant	Condition	Variant called by pipeline in mosaic parent?	Level of mosaicism (%)	Parent with mosaicism	Clinical follow‐up of parent
11	*COL1A2* NM_000089.4 c.2845G>A p.(Gly949Ser)	Osteogenesis imperfecta	No	6.77	Mother	No symptoms
12	*COL2A1* NM_001844.5 c.2365G>A p.(Gly789Ser)	*COL2A1*—related skeletal dysplasia	Yes	26.09	Mother	Short stature and chest wall deformity
13	*SOX9* NM_000346.4 c.356C>A p.(Ala119Glu)	Campomelic dysplasia	Yes	10.29	Mother	No symptoms
14	*COL2A1* NM_001844.5 c.2134_2151del p.(Ala712_Gly717del)	*COL2A1*—related skeletal dysplasia	No	6.81^a^	Father	No symptoms

^a^ Variant level in mosaic parent may be higher due to the variant being an indel and only being called at a VAF of 23.72% in the heterozygous proband.

## 4 Discussion

The cases we present here highlight the need to consider the sensitivity of the laboratory technique used when counselling parents of recurrence risk of ‘de novo’ variants. Sanger sequencing was the historic method of choice for testing parental samples and is still routinely used when singleton testing has been performed for the proband. This is despite numerous studies showing that Sanger sequencing has not detected cases of somatic mosaicism of 3.4%–10% when a more sensitive technique is used [6, 7, 8, 19, 22].

For Sanger sequencing, laboratories standardly quote a sensitivity of 10% [4, 5] for the detection of somatic mosaicism. This is supported by this study where the variant is clearly visible by Sanger sequencing when over 10%. Our data also show that when the Sanger sequencing is of good quality, variants at a lower percentage allele fraction can be detected; however, the sensitivity is not consistent. For case 7 (Figure 1C), there is no sign of a mutant peak in the mosaic parent (6.37% mosaicism) despite clean sequencing. However, in case 3 (3.88% mosaicism), a low‐level peak was observed although this had previously not been identified as mosaicism by the testing laboratory. If there is background noise in the Sanger sequencing, then the sensitivity is reduced further, as illustrated in case 4 (Figure 1B), where mosaicism was missed due to a low‐level peak also being seen in the normal control. In any case tested by Sanger sequencing, it is imperative that the laboratory quotes their determined sensitivity of the testing on the report. Parental somatic mosaicism can clearly be observed using the PCR‐NGS testing method even as low as 0.35% VAF.

For trio NGS sequencing strategies, the sensitivity for detecting parental mosaicism is dependent on both sequencing depth and the bioinformatic pipeline filtering settings. However, our study shows that manual inspection can detect mosaic variants that the pipeline has filtered out and is a recommended approach. An additional consideration is the type of variant being detected as indels have reduced sensitivity to be called from short read sequencing data due to reads that end within the indel or reduced alignment, which increases with the size of the indel. Case 14 in our study highlights this issue with the 17bp deletion only being called in 23.72% of reads in the heterozygous proband. This is also observed by others [22]. Most prenatal sequencing studies to date have been based on exome sequencing [16, 17, 19]. There is a move towards testing via genome sequencing and one of the disadvantages of this testing is the potential lower sequencing depth with subsequent decreased sensitivity to detect mosaicism [20], although some studies have successfully detected mosaicism using genome sequencing data [7]. As the depth of sequencing is not consistent for every base or across samples, laboratories should consider adding the depth of sequencing at the variant in question in the report when reporting ‘de novo’ variants to aid the clinical team in their counselling. If this is not included in the report, then it would be prudent to ask the laboratory for both the depth and the pipeline sensitivity and if manual inspection of the reads has been performed.

Detection of mosaicism in parental blood is not able to provide an accurate recurrence risk of future pregnancies. However, it does confirm that the variant must be present in germline cells due to the combination of the mosaicism and the previously affected child and therefore counselling will change. A recent study, PREGCARE [22], aimed to develop a personalised recurrence risk of parents with ‘de novo’ variants. In this study, they used deep NGS on different tissue types (buccal, blood, saliva, urine and sperm) to detect parental somatic (as well as paternal germline) mosaicism. All cases with paternal mosaicism detected in sperm had a significantly higher mutant load level than the other tissue types, suggesting that this would be the best sample (from the father) to test when determining if a variant is de novo. This also has the advantage that if mosaicism is detected, an accurate recurrence risk can be given based on the mutant VAF in the sperm sample. However, consideration must be given to the practicalities of this method as acquiring a sperm sample may be complex and many laboratories may not accept this sample type. They also recommend always testing buccal samples as well as blood from both parents since in a smaller area of tissue mosaicism is likely to be higher. Although the study findings demonstrated the utility of these methods to refine recurrence risk, the practicality and cost of these methods in publicly funded health services need to be considered. It should also be noted that the absence of the variant in blood using sensitive methods does not rule out the chance of germline mosaicism as it can still be confined to the germline (e.g., the FAM27 *MECP2* case in the PREGGCARE study) and therefore has less impact on counselling.

Our study also raises a number of counselling issues requiring consideration when referring parents thought to be at germline mosaicism risk of NIPD. Parents need to be aware that the more sensitive methods required to analyse cell free foetal DNA may detect one of the parents as a low‐level carrier with subsequent change in recurrence risks. Parents also need to be aware that with current technologies, NIPD will not be possible if maternal somatic mosaicism is detected. This may change as technologies advance.

All but two of the cases where somatic mosaicism was detected had no clinical symptoms prior to referral. The two that did have symptoms were known at the point of referral. However, a clinical review is indicated in parents identified as somatic mosaics as mutant load may vary in different tissues and there may be implications for the parents' own health.

The NIPD cohort we describe here is at known high risk because of a family history and a targeted approach to diagnosis is undertaken with the maternal sample run in parallel as somatic mosaicism in the mother has the potential to cause false positive results. Increasingly we are seeing commercial offerings of non‐invasive screening (NIPS) for monogenic conditions in pregnancies with no family history. In some cases, there is no report of parallel maternal testing which runs a risk of false positive results due to maternal mosaicism. This is further evidence supporting the necessity for appropriate follow‐up studies in cases where a foetal variant is identified.

There are some limitations to our study. Firstly, we only had access to blood samples for testing and did not have ethical approval to reconsent patients to obtain other sample types. Secondly, the PCR‐NGS method involves amplification and therefore the levels of mutant may be impacted by amplification bias and are therefore an estimate rather than an absolute percentage.

## 5 Conclusions

These cases highlight an issue that can arise during work‐up for NIPD using NGS or when offering trio sequencing for foetal diagnosis. Parents need to be aware that they may be identified as a mosaic carrier and that recurrence risks may therefore change, and in the case of maternal mosaicism, NIPD may not be straightforward or possible. We provide further evidence that Sanger sequencing is not a suitable method to detect low level mosaicism and consideration should be given to a more sensitive method to improve accuracy. This finding is not novel—it has been reported in many publications. However, Sanger sequencing remains a common method for parental testing in clinical laboratories. It also highlights the need for laboratories to provide information on NGS depth at the variant of interest in parental reports for de novo variants to allow referrers to be able to assess the sensitivity of the test used. Finally, we have demonstrated the need to test the maternal genomic DNA alongside the cell‐free DNA for all NIPD/S assays to avoid the possibility of maternal mosaicism leading to false positive results. Parents identified as mosaics should also undergo clinical evaluation since there may be implications for their own health as well as recurrence risks in future pregnancies.

## Ethics Statement

This study was a clinical audit and no identifiable data is included. As such, it did not require approval from a research ethics committee. The audit was registered with Great Ormond Street Hospital for Children NHS Foundation Trust (ref 1925 for non‐invasive prenatal diagnosis services and ref 2781 for prenatal exome sequencing). As this is a clinical service audit and no patient identifiable data is included, specific consent was not required.

## Conflicts of Interest

The authors declare no conflicts of interest.

## Supporting information

Supporting Information S1

## Data Availability

The data that support the findings of this study are available on request from the corresponding author. The data are not publicly available due to privacy or ethical restrictions.

## References

[1] R. Rahbari , A. Wuster , S. J. Lindsay , et al., “Timing, Rates and Spectra of Human Germline Mutation,” Nature Genetics 48, no. 2 (2016): 126–133, 10.1038/ng.3469. 26656846 PMC4731925

[2] H. Jonsson , P. Sulem , G. A. Arnadottir , et al., “Multiple Transmissions of De Novo Mutations in Families,” Nature Genetics 50, no. 12 (2018): 1674–1680, 10.1038/s41588-018-0259-9. 30397338

[3] C. F. Wright , E. Prigmore , D. Rajan , et al., “Clinically‐Relevant Postzygotic Mosaicism in Parents and Children With Developmental Disorders in Trio Exome Sequencing Data,” Nature Communications 10, no. 1 (2019): 2985, 10.1038/s41467-019-11059-2. PMC6611863 31278258

[4] X. Xu , X. Yang , Q. Wu , et al., “Amplicon Resequencing Identified Parental Mosaicism for Approximately 10% of ‘De Novo’ SCN1A Mutations in Children With Dravet Syndrome,” Human Mutation 36, no. 9 (2015): 861–872, 10.1002/humu.22819. 26096185 PMC5034833

[5] Z. Chen , K. Moran , J. Richards‐Yutz , et al., “Enhanced Sensitivity for Detection of Low‐Level Germline Mosaic RB1 Mutations in Sporadic Retinoblastoma Cases Using Deep Semiconductor Sequencing,” Human Mutation 35, no. 3 (2014): 384–391, 10.1002/humu.22488. 24282159 PMC4112364

[6] C. J. Brewer , M. Gillespie , J. Fierro , et al., “The Value of Parental Testing by Next‐Generation Sequencing Includes the Detection of Germline Mosaicism,” Journal of Molecular Diagnostics 22, no. 5 (2020): 670–678, 10.1016/j.jmoldx.2020.02.001. 32092540

[7] C. B. Cook , L. Armstrong , C. F. Boerkoel , et al., “Somatic Mosaicism Detected by Genome‐Wide Sequencing in 500 Parent‐Child Trios With Suspected Genetic Disease: Clinical and Genetic Counseling Implications,” Cold Spring Harbor Molecular Case Studies 7, no. 6 (2021): a006125, 10.1101/mcs.a006125. 34697084 PMC8751411

[8] L. Qin , J. Wang , X. Tian , et al., “Detection and Quantification of Mosaic Mutations in Disease Genes by Next‐Generation Sequencing,” Journal of Molecular Diagnostics 18, no. 3 (2016): 446–453, 10.1016/j.jmoldx.2016.01.002. 26944031

[9] R. Mellis , N. Chandler , and L. S. Chitty , “Next‐Generation Sequencing and the Impact on Prenatal Diagnosis,” Expert Review of Molecular Diagnostics 18, no. 8 (2018): 689–699, 10.1080/14737159.2018.1493924. 29962246

[10] L. S. Chitty , S. Mason , A. N. Barrett , et al., “Non‐Invasive Prenatal Diagnosis of Achondroplasia and Thanatophoric Dysplasia: Next‐Generation Sequencing Allows for a Safer, More Accurate, and Comprehensive Approach,” Prenatal Diagnosis 35, no. 7 (2015): 656–662, 10.1002/pd.4583. 25728633 PMC4657458

[11] L. A. Jenkins , Z. C. Deans , C. Lewis , and S. Allen , “Delivering an Accredited Non‐Invasive Prenatal Diagnosis Service for Monogenic Disorders and Recommendations for Best Practice,” Prenatal Diagnosis 38, no. 1 (2018): 44–51, 10.1002/pd.5197. 29266293

[12] R. Mellis , N. Chandler , L. Jenkins , and L. S. Chitty , “The Role of Sonographic Phenotyping in Delivering an Efficient Noninvasive Prenatal Diagnosis Service for FGFR3‐Related Skeletal Dysplasias,” Prenatal Diagnosis 40, no. 7 (2020): 785–791, 10.1002/pd.5687. 32227640

[13] M. Hill , P. Twiss , T. I. Verhoef , et al., “Non‐Invasive Prenatal Diagnosis for Cystic Fibrosis: Detection of Paternal Mutations, Exploration of Patient Preferences and Cost Analysis,” Prenatal Diagnosis 35, no. 10 (2015): 950–958, 10.1002/pd.4585. 25708280 PMC4672687

[14] N. J. Chandler , H. Ahlfors , S. Drury , et al., “Non‐Invasive Prenatal Diagnosis for Cystic Fibrosis: Implementation, Uptake, Outcome, and Implications,” Clinical Chemistry 66, no. 1 (2020): 207–216, 10.1373/clinchem.2019.305011. 31551312

[15] E. Preka , D. Ellershaw , N. Chandler , et al., “Cell‐Free DNA in Pediatric Solid Organ Transplantation Using a New Detection Method of Separating Donor‐Derived From Recipient Cell‐Free DNA,” Clinical Chemistry 66, no. 10 (2020): 1300–1309, 10.1093/clinchem/hvaa173. 32882007

[16] N. J. Chandler , E. Scotchman , R. Mellis , V. Ramachandran , R. Roberts , and L. S. Chitty , “Lessons Learnt From Prenatal Exome Sequencing,” Prenatal Diagnosis 42, no. 7 (2022): 831–844, 10.1002/pd.6165. 35506549 PMC9325487

[17] T. Gambin , Q. Liu , J. A. Karolak , et al., “Low‐Level Parental Somatic Mosaic SNVs in Exomes From a Large Cohort of Trios With Diverse Suspected Mendelian Conditions,” Genetics in Medicine 22, no. 11 (2020): 1768–1776, 10.1038/s41436-020-0897-z. 32655138 PMC7606563

[18] Y. Cao , M. J. Tokita , E. S. Chen , et al., “A Clinical Survey of Mosaic Single Nucleotide Variants in Disease‐Causing Genes Detected by Exome Sequencing,” Genome Medicine 11, no. 1 (2019): 48, 10.1186/s13073-019-0658-2. 31349857 PMC6660700

[19] D. D. Domogala , T. Gambin , R. Zemet , et al., “Detection of Low‐Level Parental Somatic Mosaicism for Clinically Relevant SNVs and Indels Identified in a Large Exome Sequencing Dataset,” Human Genomics 15, no. 1 (2021): 72, 10.1186/s40246-021-00369-6. 34930489 PMC8686574

[20] I. Miceikaite , C. Fagerberg , C. Brasch‐Andersen , et al., “Comprehensive Prenatal Diagnostics: Exome Versus Genome Sequencing,” Prenatal Diagnosis 43, no. 9 (2023): 1132–1141, 10.1002/pd.6402. 37355983

[21] G. England , PaneApp UK: Fetal Anomalies Panel, https://panelapp.genomicsengland.co.uk/panels/478/.

[22] M. Bernkopf , U. B. Abdullah , S. J. Bush , et al., “Personalized Recurrence Risk Assessment Following the Birth of a Child With a Pathogenic De Novo Mutation,” Nature Communications 14, no. 1 (2023): 853, 10.1038/s41467-023-36606-w. PMC9932158 36792598

