# Supplementary material for: Parental Somatic Mosaicism Detected During Prenatal Diagnosis
Source: Prenat Diagn. 2024 Nov 25;45(2):171–7. doi: 10.1002/pd.6712 (PMC11790521; doi:10.1002/pd.6712)
Supplement: Supplementary file 1 — Supporting Information S1 [file PD-45-171-s001.docx]

Supplementary data

**Assessing background levels and lower limit of detection**

To assess the accuracy of our method DNA mixes were made to represent artificial samples using known concentrations of samples from different unrelated patients to create 5%, 2%, 1%, 0.5% and 0.25% chimeric DNA samples. These were tested for 50 common single nucleotide polymorphisms (SNPs). These artificial samples were run alongside the original genomic DNA to allow us to determine major and minor sample genotypes. To determine the mean background level of counts, SNPs homozygous for the same allele in both genotypes were examined; any counts for the alternate allele represent technical background either from PCR or sequencing errors. The mean background minor allele frequency was 0.12%. To determine the lower limit of detection we used SNPs where the major genotype was homozygous and minor genotype heterozygous, of which we had 6. All 6 SNPs were correctly called in the 1%, 2% and 5% artificial chimeric samples. For both the 0.5% and 0.25% samples 1 SNP was incorrectly classified as detected (see supplementary table 1).

We have therefore set our threshold for calling a variant as present at 1% to prevent incorrect results. Each case was also run twice to assess reproducibility, with normal controls run to measure variant background of each specific assay. The background level in the normal control had to be lower than the 0.12% threshold for us to call the variant as detected in the parental sample.

Supplementary table 1- limit of detection testing results for artificial chimeric samples of varying percentages.

| **Chimeric sample %** | **5%** | **2%** | **1%** | **0.50%** | **0.25%** |
| --- | --- | --- | --- | --- | --- |
| Average counts per SNP | 10580 | 9559 | 17994 | 10062 | 14873 |
| **Total informative SNPs** | **6** | **6** | **6** | **6** | **6** |
| Correctly called | 6 | 6 | 6 | 5 | 5 |
| Incorrectly called | 0 | 0 | 0 | 1 | 1 |
| **Average minor allele %** | **6.73** | **3.07** | **1.29** | **0.79** | **0.28** |


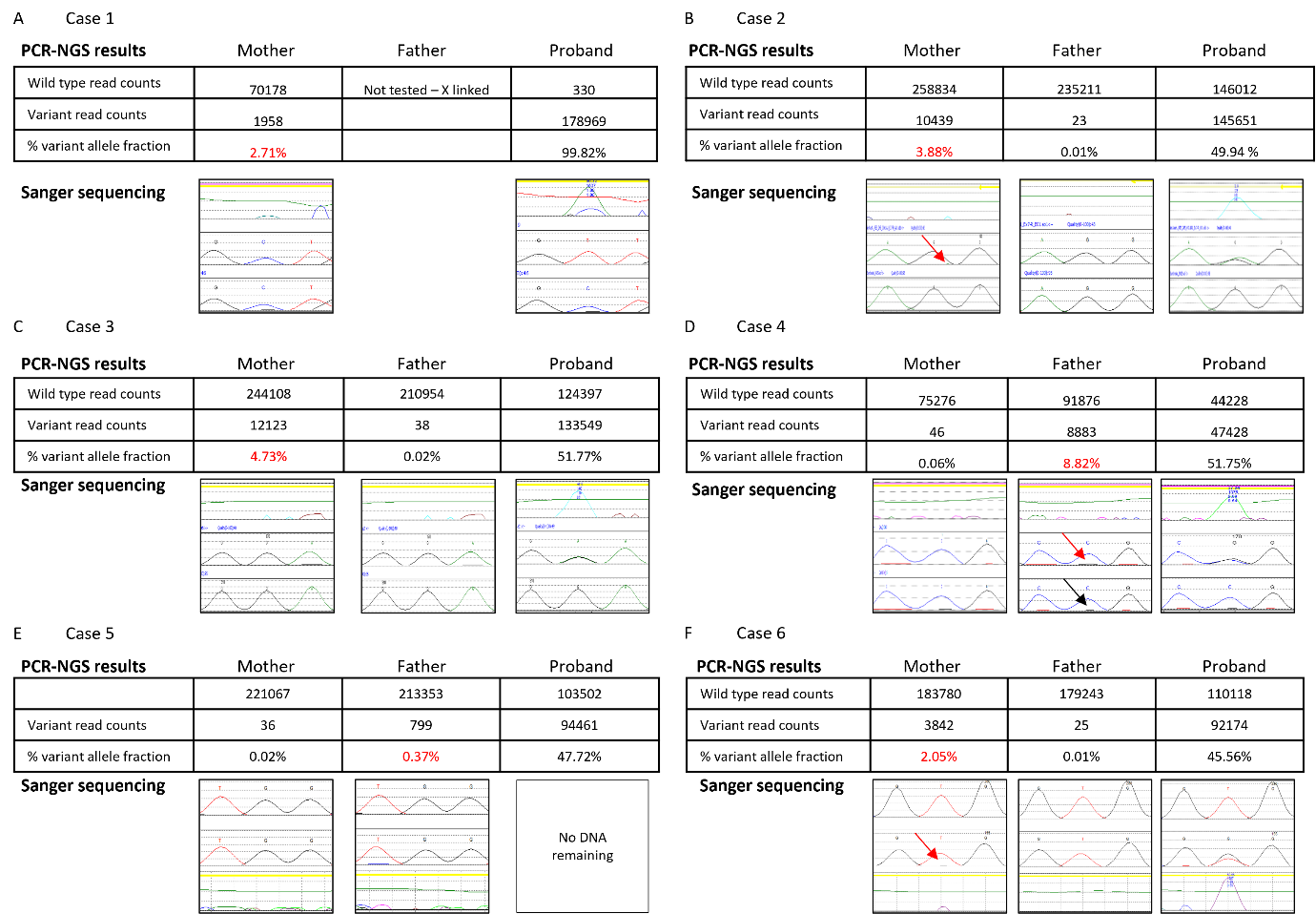
 Supplementary figure 1: A comparison of Sanger sequencing and PCR capture next generation sequencing results of parental and affected proband genomic DNA sample for cases 1-6 (A-F) where somatic mosaicism was not known about prior to NIPD assay development. For the Sanger sequencing traces all variants occur at the middle peak in each image. The middle panel in each figure shows traces from the sample tested, the other sequence trace is from a normal control sample (either top or bottom depending on if forward or reverse) and the other panel is the electropherogram traces comparing the peaks detected in the sample trace in comparison to the normal control. Red arrows indicate signs of mosaicism, black arrows indicate background noise causing mosaicism to be missed. PCR capture Next Generation Sequencing results are given as the number of reads for the wild type and variant sequences. The percentage VAF has also been calculated with the values in red indicating somatic mosaicism.


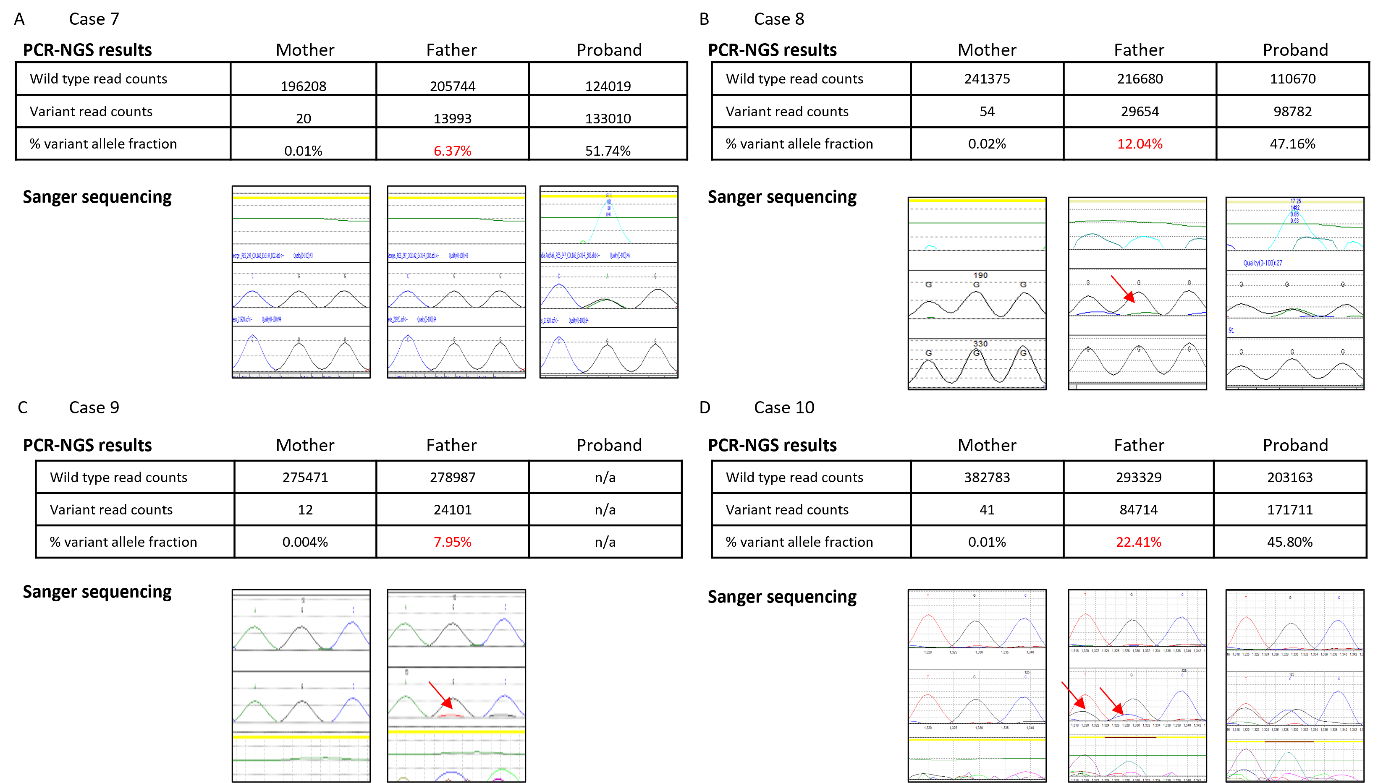
 Supplementary figure 2: A comparison of Sanger sequencing and PCR capture Next Generation Sequencing results of parental and affected proband genomic DNA sample for cases 7-10 (A-D) where somatic mosaicism was known about prior to NIPD assay development. For the Sanger sequencing traces all variants occur at the middle peak in each image. The middle panel in each figure shows traces from the sample tested, the other sequence trace is from a normal control sample (either top or bottom depending on if forward or reverse) and the other panel is the electropherogram traces comparing the peaks detected in the sample trace in comparison to the normal control. Red arrows indicate signs of mosaicism, black arrows indicate background noise causing mosaicism to be missed. PCR capture Next Generation Sequencing results are given as the number of reads for the wild type and variant sequences. The percentage VAFs have also been calculated with the values in red indicating somatic mosaicism.


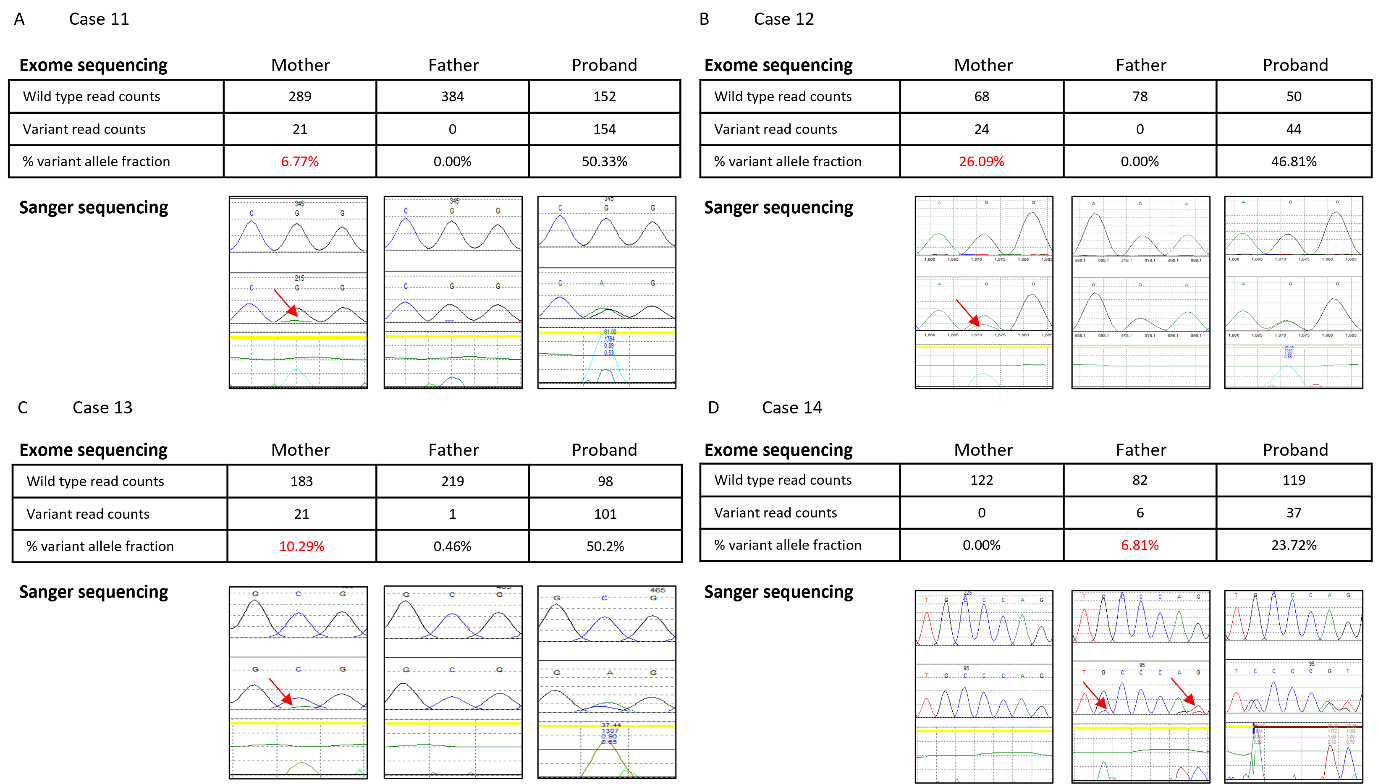
 Supplementary figure 3: Trio exome sequencing results and Sanger sequencing traces of parental and affected proband genomic DNA sample for cases 11-14 (A-D). Exome sequencing results are given as the number of reads for the wild type and variant sequences. The percentage VAFs have also been calculated with the values in red indicating somatic mosaicism. For the Sanger sequencing, the top panel is sequence traces from a normal control sample, middle panel traces from the sample tested and the bottom panel is the electropherogram traces comparing the peaks detected in the sample trace in comparison to the normal control. Red arrows indicate signs of mosaicism.

Proband:


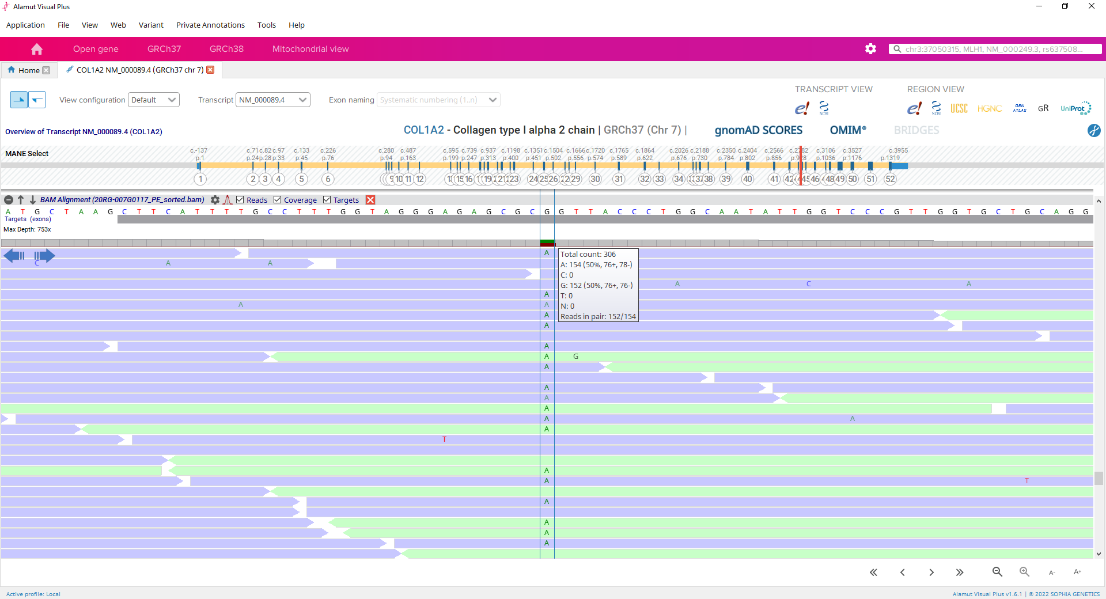


Mother:


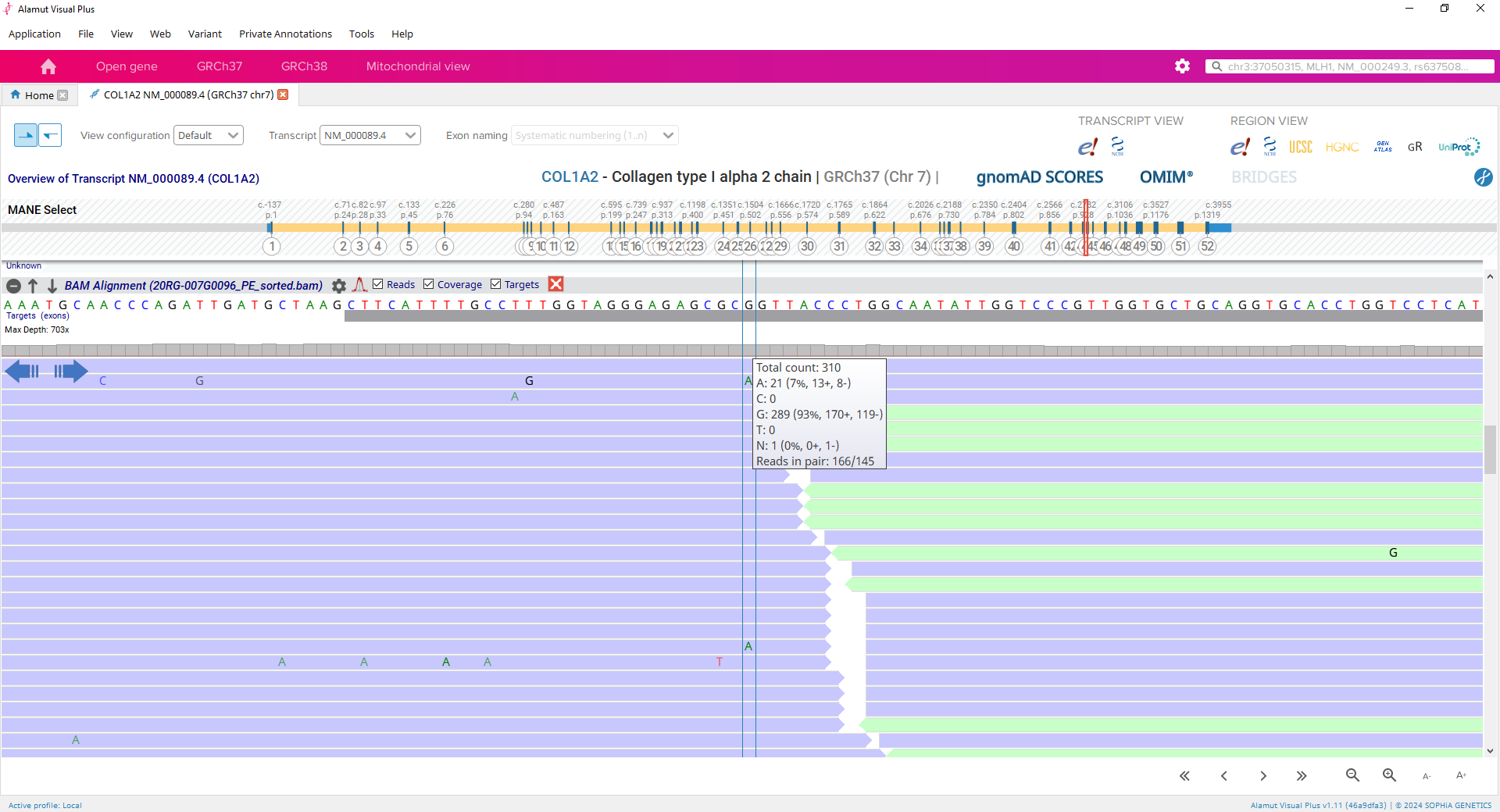


Father:


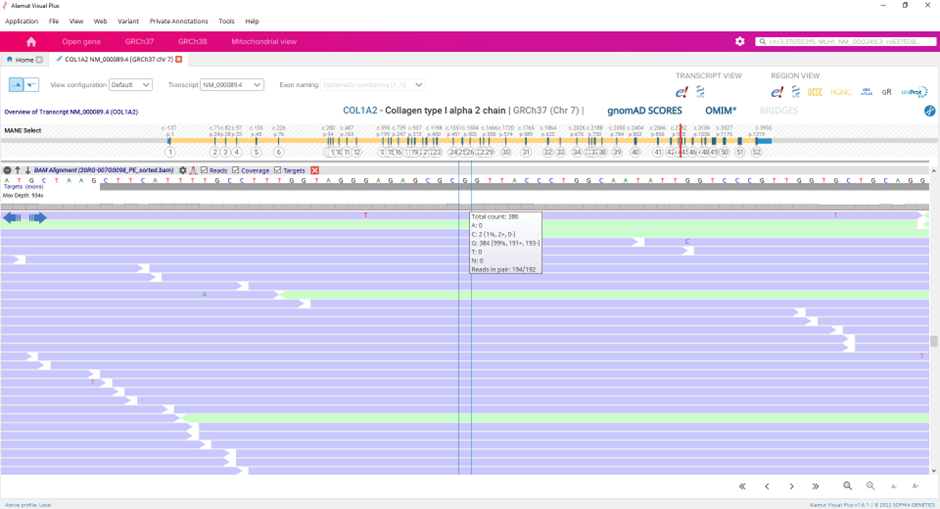
]

Supplementary figure 4: BAM alignment files for case 11 *COL1A2* NM_000089.3 c.2845G>A p.(Gly949Ser). The reference sequence (according to GRCh37) is shown above the targets. The sequencing reads are indicated by the green (forward reads) and blue (reverse reads). Where differences between the reference sequence and the sequencing read are present this is indicated by the letter of the alternative nucleotide called. The position of the variant of interest is indicated by the blue lines and the number and percentage of reads with each nucleotide is shown in the text box.


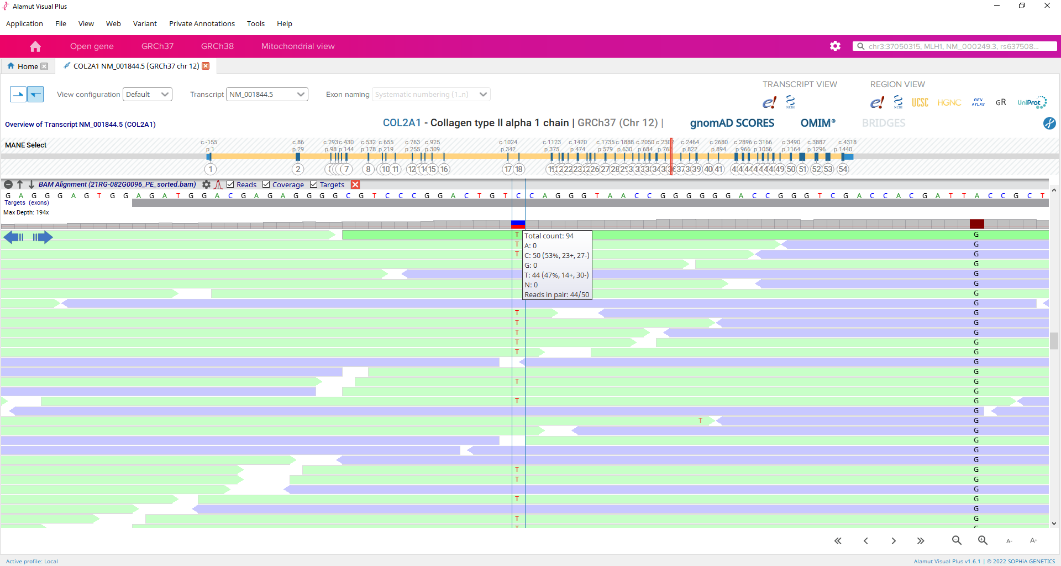
Proband:


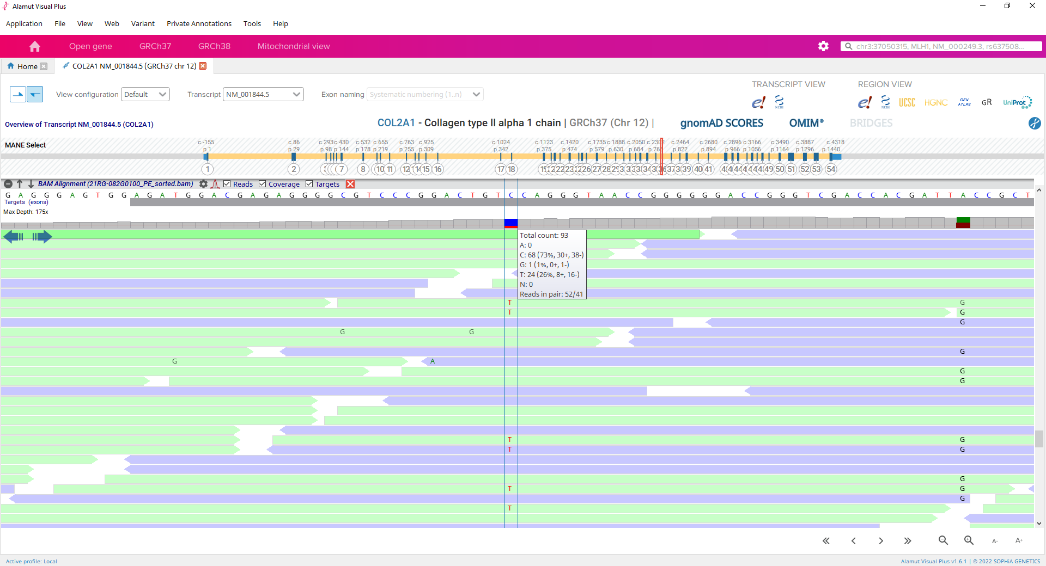
Mother:


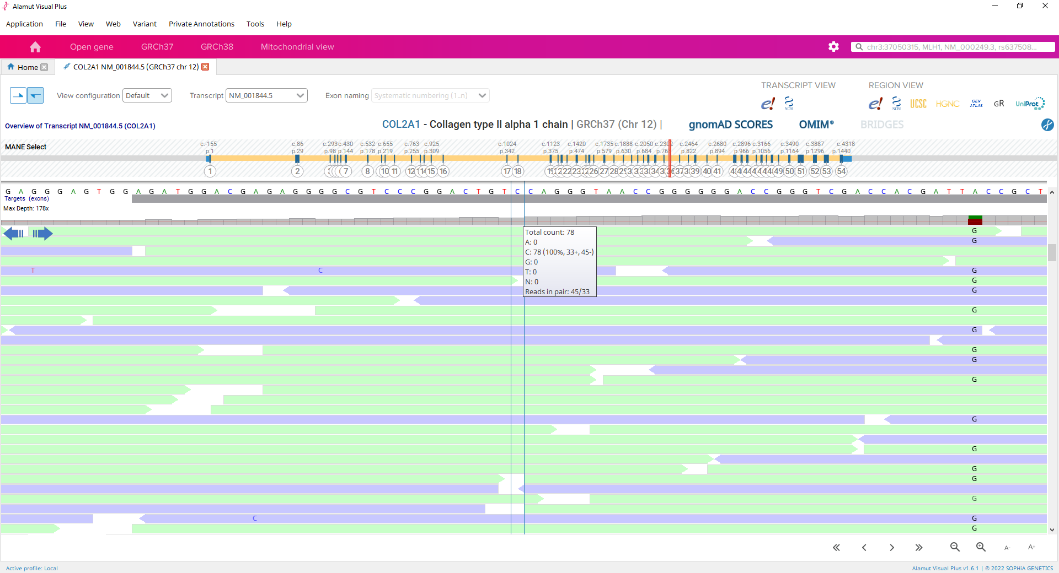
Father:

Supplementary figure 5: BAM alignment files for case 12 *COL2A1* NM_001844.5 c.2365G>A p.(Gly789Ser). The reference sequence (according to GRCh37) is shown above the targets. The sequencing reads are indicated by the green (forward reads) and blue (reverse reads). Where differences between the reference sequence and the sequencing read are present this is indicated by the letter of the alternative nucleotide called. The position of the variant of interest is indicated by the blue lines and the number and percentage of reads with each nucleotide is shown in the text box.

Proband:


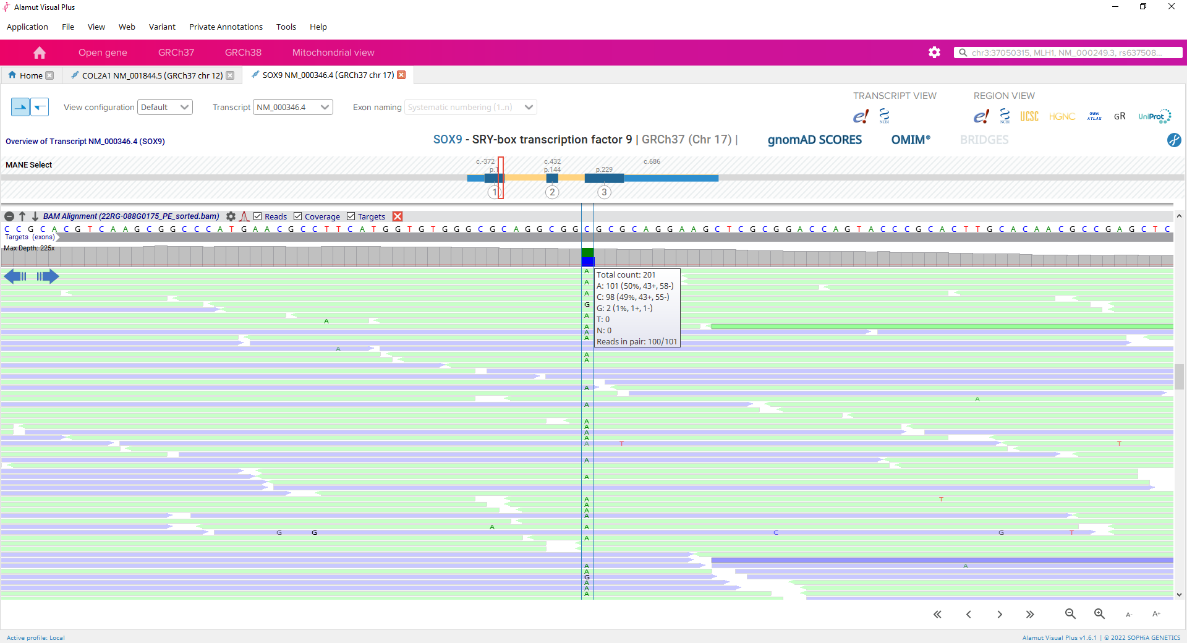


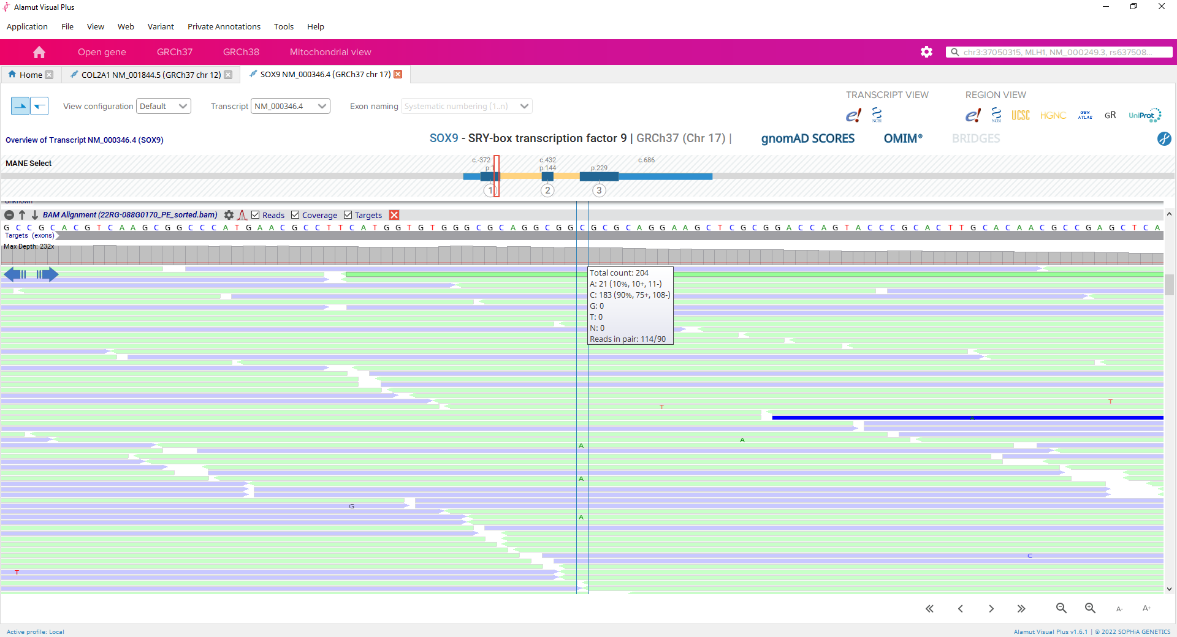
Mother:


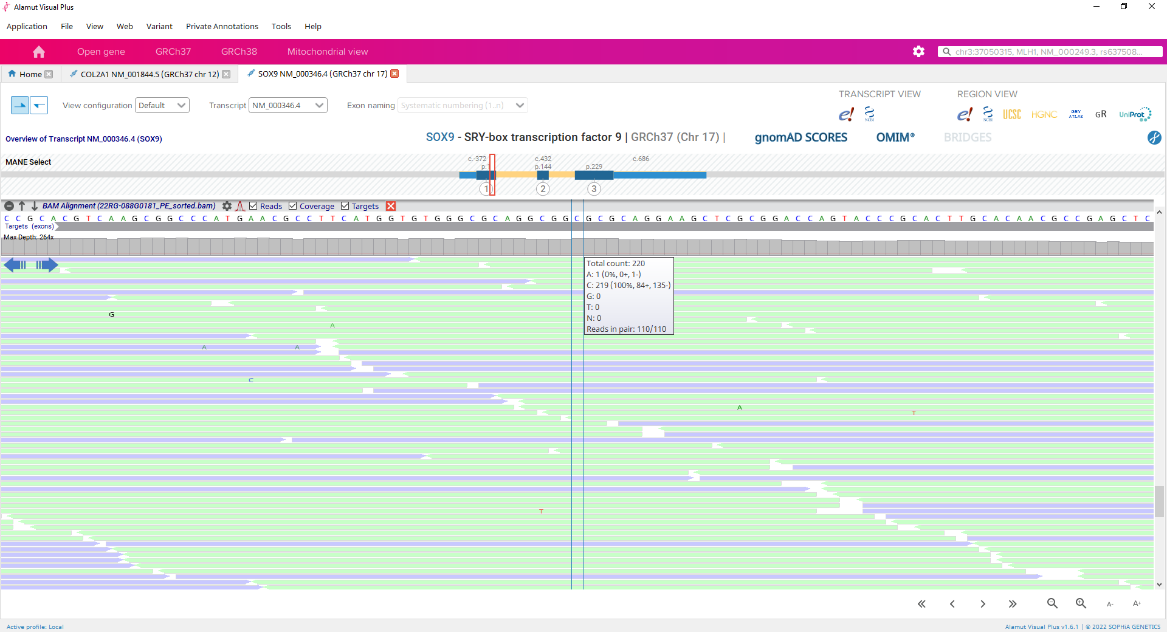
Father:

Supplementary figure 6: BAM alignment files case 13 *SOX9* NM_000346.4: c.356C>A p.(Ala119Glu). The reference sequence (according to GRCh37) is shown above the targets. The sequencing reads are indicated by the green (forward reads) and blue (reverse reads). Where differences between the reference sequence and the sequencing read are present this is indicated by the letter of the alternative nucleotide called. The position of the variant of interest is indicated by the blue lines and the number and percentage of reads with each nucleotide is shown in the text box.

Proband:
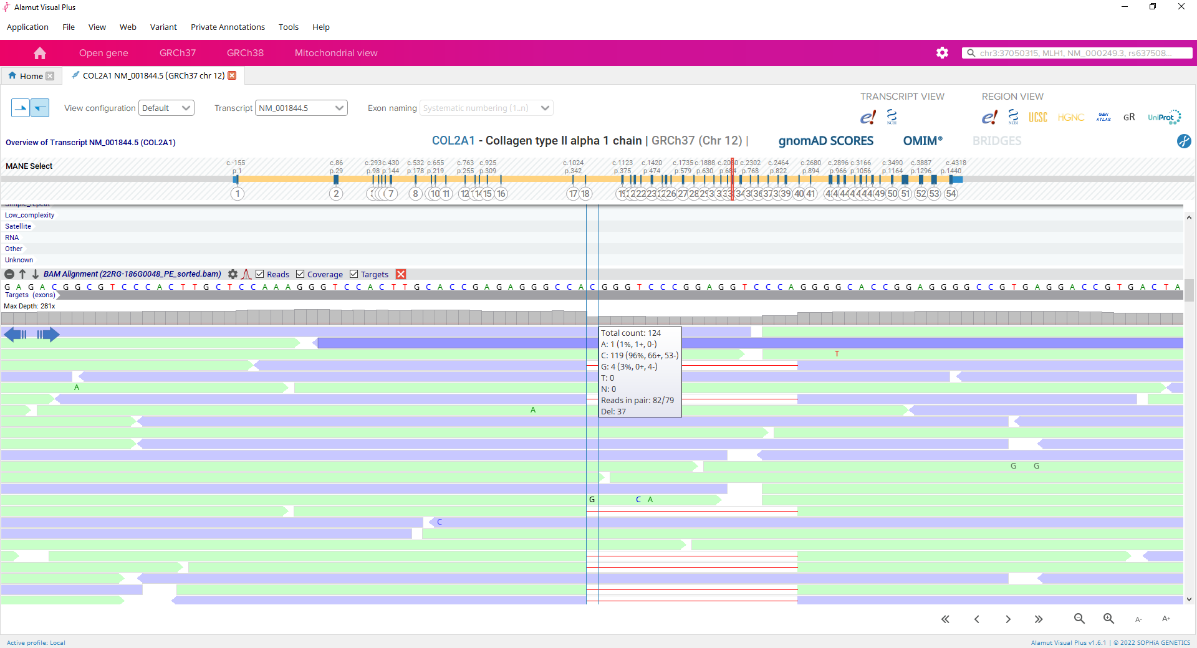


Mother:
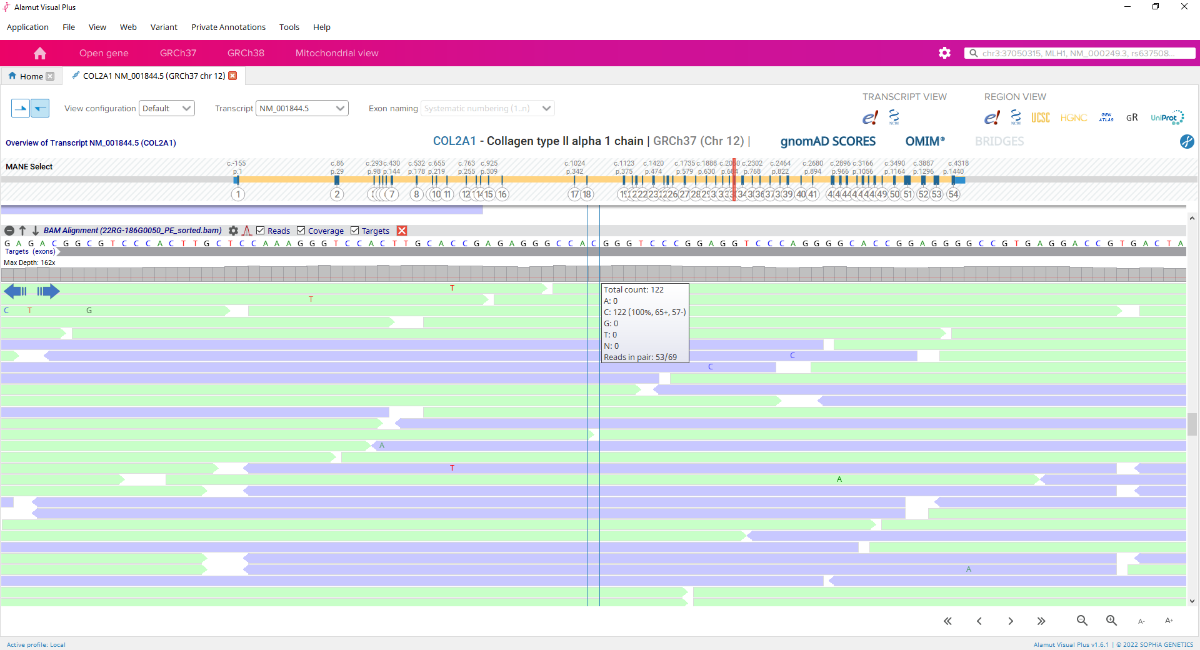


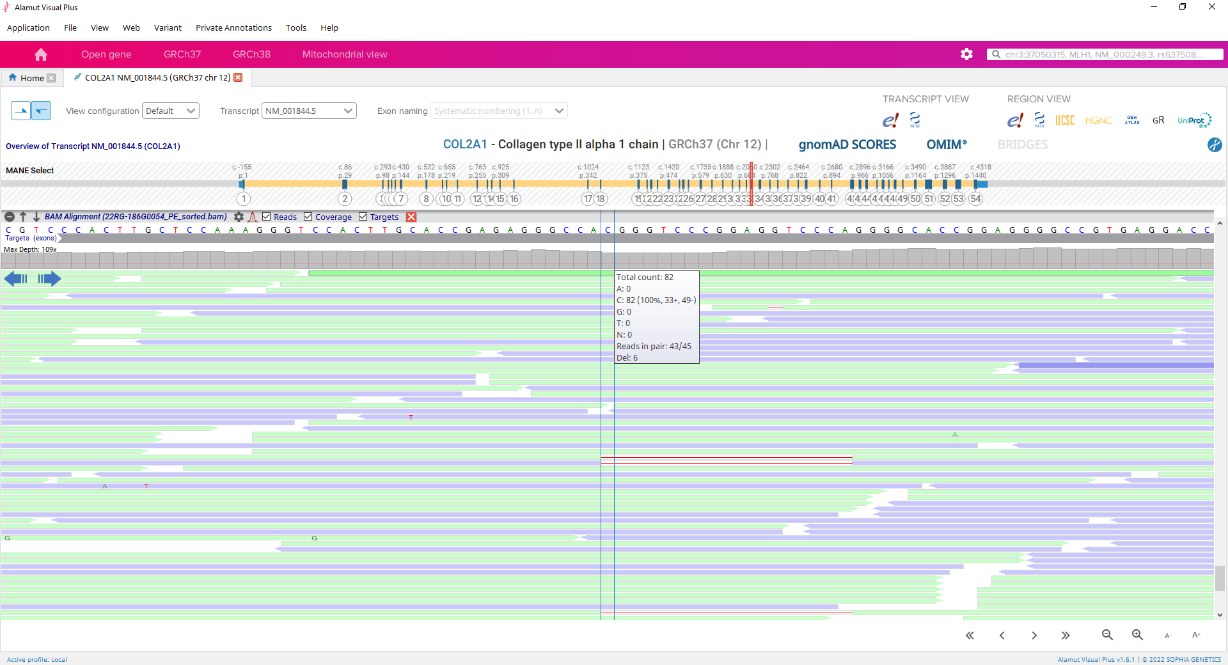
Father:

Supplementary figure 7: BAM alignment files case 14 *COL2A1* NM_001844.5 c.2134_2151del p.(Ala712_Gly717del). The reference sequence (according to GRCh37) is shown above the targets. The sequencing reads are indicated by the green (forward reads) and blue (reverse reads). Where differences between the reference sequence and the sequencing read are present this is indicated by the letter of the alternative nucleotide called or a read line indicating the nucleotide is not present (deleted). The position of the variant of interest is indicated by the blue lines and the number and percentage of reads with each nucleotide is shown in the text box.
